# A Composite DC–DC Converter Based on the Versatile Buck–Boost Topology for Electric Vehicle Applications

**DOI:** 10.3390/s22145409

**Published:** 2022-07-20

**Authors:** Catalina González-Castaño, Carlos Restrepo, Freddy Flores-Bahamonde, Jose Rodriguez

**Affiliations:** 1 Department of Engineering Sciences, Universidad Andres Bello, Santiago 7500971, Chile; catalina.gonzalez@unab.cl (C.G.-C.); freddy.flores@unab.cl (F.F.-B.); 2 Department of Electromechanics and Energy Conversion, Universidad de Talca, Curicó 3340000, Chile; 3 Department of Engineering, Universidad San Sebastián, Santiago 8420524, Chile; jose.rodriguezp@uss.cl

**Keywords:** electric vehicles, dc–dc power converters, digital control

## Abstract

The composite converter allows integrating the high-efficiency converter modules to achieve superior efficiency performance, becoming a prominent solution for electric transport power conversion. In this work, the versatile buck–boost dc–dc converter is proposed to be integrated into an electric vehicle composite architecture that requires a wide voltage range in the dc link to improve the electric motor efficiency. The inductor core of this versatile buck–boost converter has been redesigned for high voltage applications. The versatile buck–boost converter module of the composite architecture is in charge of the control stage. It provides a dc bus voltage regulation at a wide voltage operation range, which requires step-up (boost) and step-down (buck) operating modes. The PLECS thermal simulation of the composite architecture shows a superior power conversion efficiency of the proposed topology over the well-known classical noninverting buck–boost converter under the same operating conditions. The obtained results have been validated via experimental efficiency measures and experimental transient responses of the versatile buck–boost converter. Finally, a hardware-in-the-loop (HIL) real-time simulation system of a 4.4 kW powertrain is presented using a PLECS RT Box 1 device. The HIL simulation results verified the accuracy of the theoretical analysis and the effectiveness of the proposed architecture.

## 1. Introduction

In recent years, different efforts have been undertaken to reduce GHG emissions and improve the performance of the transportation sector, employing the development of new fuels and the electrification of transport [1]. The latter is a promising approach with potential benefits in improving energy security by diversifying the energy sources and fostering economic growth by creating new advanced industries focused on vehicular technologies such as battery electric vehicles (BEVs), hybrid electric vehicles (HEVs), and hydrogen fuel-cell electric vehicles (HFCEVs). Most importantly, its technology is environment friendly since renewable energy is integrated into the power system [1,2,3].

**Figure 1:**
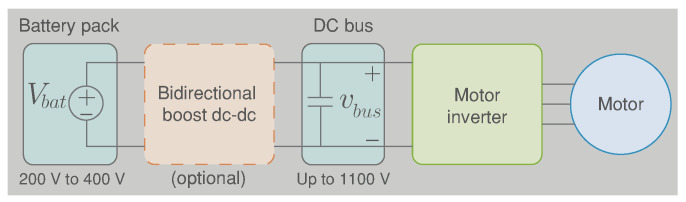
Typical electric vehicle powertrain architecture.

**Figure 2:**
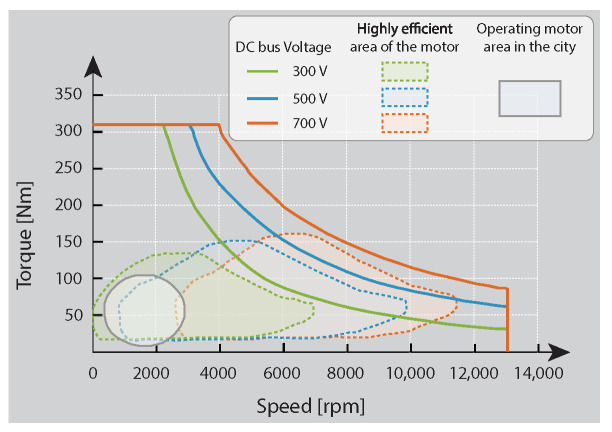
The high-efficiency operation area of the motor in a Honda Accord 2014 PHEV under different dc bus voltages [13].

A typical electric vehicle powertrain architecture includes a battery pack, a motor drive (motor inverter and electric motor), and sometimes a bidirectional dc–dc power converter between the battery and the inverter converter as shown in Figure 1 [4]. The use of this optional converter is justified in each of the components that form the system as described below:

- Battery system: Battery cells for EVs are usually connected in series to meet the voltage requirements of the power inverter. However, this connection exponentially increases the probability of failure of the battery pack [5]. In addition, the performance of the whole battery pack is limited by the weakest cell, which requires an oversizing of the power inverter and the electric motor to ensure peak power delivery at the lowest and the highest state of charge (SoC) of the battery pack [5,6]. Thus, a boost dc–dc converter optimises the battery size, avoiding oversizing the motor drive, which results in a cost reduction [4,7]. To increase the use of EVs, many countries have introduced fuel economy regulations. Historically, the average EV lithium-ion battery price dropped from USD 800/kWh in 2011 to roughly USD 300/kWh in 2016. The battery costs at pack level for an Audi e-tron is USD 157/kWh in 2019. Therefore, further cost reductions are predicted to be down to USD 76/kWh by 2030 [8,9].
- Power inverter: A power inverter directly connected to the battery system does not have the same performance at all modulation indices (MI), and it is more efficient and produces better waveforms at higher MI values. When the motor reaches the base speed, the motor phase voltage remains constant using an MI=1 which produces an efficient operation of the inverter [10]. Nevertheless, for values below the base speed (urban driving), the phase voltage of the motor is increased proportionally to the speed, which produces an inverter operation in low-efficiency zones (MI less than 1). Thus, a boost dc–dc converter is used to control the voltage at the input of the inverter according to the motor speed and optimize the efficiency of the inverter (high values of 1) in a wider range of operating speeds [10].
- Electric motor: The most-used traction motors in commercial EVs are the induction machine (IM) and the permanent magnet synchronous machine (PMSM) [11,12].All these machines in the EV applications have been designed to exhibit torque/power-speed characteristics, as shown in Figure 2 [11,13]. This figure shows that the motor operating zone with high efficiency at high speeds is achieved with high voltage on the dc bus. However, with this dc bus voltage, the motor would not be operating in an efficient area like the low speeds reached in the city. Thus, the motor dynamic is governed by the input voltage and current of the inverter. Still, if the inverter is directly connected to a battery, its input voltage will be variable and not controlled. Hence, with a dc–dc converter between the battery and the inverter, the dc voltage bus in the inverter input could be optimized based on the motor speed to maximize the electric motor efficiency [12,14]. The costs of electric motors depend on the nominal power; in general, the cost for a PSM is USD 10/kWh and for a IM is USD 8/kWh [9].

Despite all the advantages of incorporating a dc–dc converter in the powertrain and the wide use in the EV market [15], there are still challenges where the improvement of power conversion efficiency stands out. In this application, the traditional dc–dc boost converter’s efficiency is poor when operating with high voltage-conversion ratios due to the high capacitor RMS current and the large semiconductor voltage and current stress [16]. This converter has low efficiency and power density at light load power, as with urban driving [17].

The efficiency improvement of the dc–dc converter has been the subject of extensive research in recent years. As a result, it has reached a high level of development to enhance the performance of automotive traction systems. Novel approaches to improve the efficiency of the dc–dc converter are proposed in the literature. Some of the most distinctive strategies are the use of soft switching [18,19,20,21], a coupled inductor, which reduces the magnetic losses and the switching losses [22,23], and multilevel converter topologies, which lead to the reduction of the voltage rating of the switches, allowing the use of MOSFETs devices instead of IGBTs. The MOSFETs devices allow higher switching frequency operation, representing a substantial reduction in the inductor size and losses while ensuring low conduction and switching losses [17,24].

**Figure 3:**
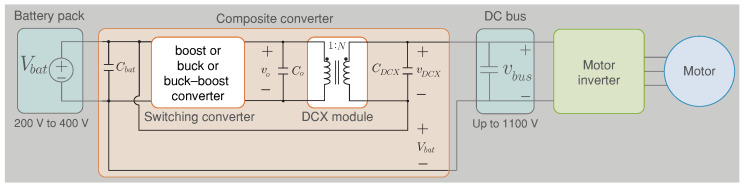
Electric vehicle powertrain architecture based on the boost composite converter A.

However, the previously proposed efficiency improvement approaches do not seek to enhance specific operating ranges’ efficiency. They have limited improvements in size, cost, and efficiency trade-offs, unlike the composite converter shown in Figure 3 [16]. In a composite converter, dissimilar smaller converter modules (switching converter(s) and an unregulated “dc transformer” DCX) are combined to process the total system power, with effective utilization of the semiconductor and reactive elements in each module to achieve superior performance of the whole system [25].

A composite converter architecture was proposed for the first time in [16] to improve the electric vehicle powertrain efficiency over a wide range of operating conditions. Furthermore, four different composite architectures were proposed in [4], and this paper focuses on the named topology A (see Figure 3) as the initial work. Moreover, the voltage stresses are shared between series-connected modules, enabling the use of lower-voltage semiconductors, having higher switching frequency and lower forward voltage drop. This composite architecture allows a new degree of freedom in power component optimization and control strategy to address the loss mechanisms associated with indirect power conversion over wide ranges of operating conditions and achieve superior efficiency as shown in Figure 2.

**Figure 4:**
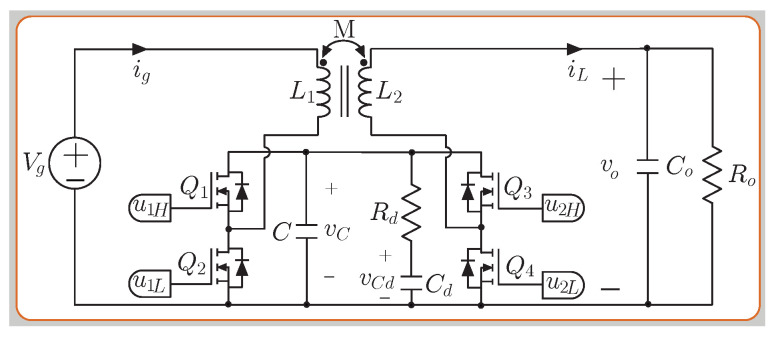
Schemes of the noninverting buck–boost versatile (BBV) converter.

The novelty of this work is integrating the versatile buck–boost converter (see Figure 4) into the EV composite converter architecture. The selected converter topology has many advantages, such as a noninverting voltage step-up and step-down characteristic, high efficiency, wide bandwidth [26], and the regulation of input or output currents because of their low ripple values [27]. This converter was initially designed for low voltage and hard-switching applications. However, a new bidirectional converter proposed for high-voltage applications is presented in [28] and for a typical electric vehicle powertrain architecture in [29] as shown in Figure 4. The main goal of this work is to study whether all the abovementioned features of the versatile buck–boost converter can be extended to a composite converter architecture, enhancing the advantages of this system. The presented study includes thermal simulation, experimental validation, and hardware-in-the-loop (HIL) real-time simulation, which has proven to be a handy tool in many applications [30,31,32,33,34,35,36,37]. Based on this state-of-the-art review, the main contributions of this paper are:

- It is the first time that the versatile buck–boost converter is used in an EV composite architecture. The proposed converter has many outstanding advantages, making it attractive to be integrated into a composite converter architecture, and its potential in EV applications has been demonstrated.
- A wide conversion ratio is achieved with very high performance in the whole operation range, ensuring an efficient regulation of the dc-bus.
- Fair efficiency comparison between the proposed versatile buck–boost converter and a classic buck–boost converter validated an improvement in all operating points of up to 6%.
- The system’s controllability is verified using challenging tests to reproduce battery voltage variations and load changes in agreement with the theoretical predictions.

This paper is organized as follows: in Section 2, the analysis of the dc–dc coupled inductors buck–boost converter is presented. Section 3 presents a detailed description of the most relevant aspects of the proposed control technique for the composite converter. Experimental, simulation, and hardware in the loop results are presented and discussed in Section 4. Finally, the main conclusions and the remaining challenges for future works are summarized in Section 5.

## 2. Analysis of Noninverting Buck–Boost Versatile Converter

The topology used for the composite architecture’s controlled module corresponds with the noninverting buck–boost versatile (BBV) converter seen in Figure 4. This section addresses a BBV converter analysis to find the inductor current slope expressions used for the current control design in each operation mode (boost or buck). The BBV converter has four MOSFETs, an $R_{d}C_{d}$ damping network, and an intermediate capacitor *C* (see Figure 4). The coupled inductor possesses a unitary ideal turns ratio N2/N1, a coupled coefficient k=0.5, a mutual inductance *M*, and equal values for the primary ($L_{1}$) and secondary ($L_{2}$) self-inductances ($L=L_{1}=L_{2}$). The converter model using the state–space averaging (SSA) method is presented in [28]. In the analysis, the use of the state–space averaging (SSA) method to model the converter leads to the following set of differential equations:

(1) $$\frac{di_{g}\left(t\right)}{dt}=\frac{L\left(V_{g}-v_{c}\left(1-u_{1L}\right)\right)-M\left(v_{o}-v_{c}u_{2H}\right)}{L^{2}-M^{2}}$$

(2) $$\frac{di_{L}\left(t\right)}{dt}=\frac{M\left(V_{g}-v_{c}\left(1-u_{1L}\right)\right)-L\left(v_{o}-v_{c}u_{2H}\right)}{L^{2}-M^{2}}$$

(3) $$\frac{dv_{c}\left(t\right)}{dt}=\frac{1}{C}\left(-i_{L}u_{2H}+i_{g}\left(-u_{1L}+1\right)-\frac{1}{R_{d}}\left(v_{c}-v_{cd}\right)\right)$$

(4) $$\frac{dv_{cd}\left(t\right)}{dt}=\frac{v_{c}-v_{cd}}{C_{d}R_{d}}$$

(5) $$\frac{dv_{o}\left(t\right)}{dt}=\frac{i_{L}}{C_{o}}-\frac{v_{o}}{R_{o}C_{o}}$$

In Figure 4, $v_{cd}$ is the voltage of the damping capacitor, $v_{c}$ is the voltage of the intermediate capacitor, $v_{o}$ is the output voltage, $i_{L}$ is the output current, $i_{g}$ is the input current, and $V_{g}$ is the input voltage. The method to select the value for the components $C_{d}$, *C*, and $R_{d}$ is presented in [28]. The selection of the intermediate capacitor size ensures adequate and robust damping of the internal dynamics; the expression corresponds to:

(6) $$R_{d}\approx 0.65\sqrt{\frac{M}{C}},\;C_{d}\geq 8C.$$

The power loss in $R_{d}$ ($P_{Rd}$) is given by the rms value of the voltage peak-to-peak ripple in the intermediate capacitor $\Delta v_{c}$ [26], as follows

(7) $$P_{Rd}=\frac{\Delta v_{c}^{2}}{12R_{d}}$$

Table 1 lists the expressions to calculate current and voltage peak-to-peak ripple, where *T* is the switching period, and $D_{1}$ and $D_{2}$ are the steady-state duty cycles in boost and in buck mode. The steps to calculate the component values of the versatile buck–boost converter are shown in Algorithm 1.
**Algorithm 1:** BBV converter design procedure **Input**: $\Delta i_{L}$, $P_{Rd}$, $V_{o}$, $V_{g}$, *T*, $I_{L}$ and $\Delta v_{c}$. **Output**: *M*, *C*, $C_{d}$ and $R_{d}$._1_ Find $R_{d}$ from Equation (7)._2_ Find *C* from equation $\Delta v_{c}$ in Table 1._3_ Compute *M* and $C_{d}$ from Equation (6)._4_ Find *L* form equation $\Delta i_{L}$ in Table 1.

## 3. Control Design of the Composite Converter

**Figure 5:**
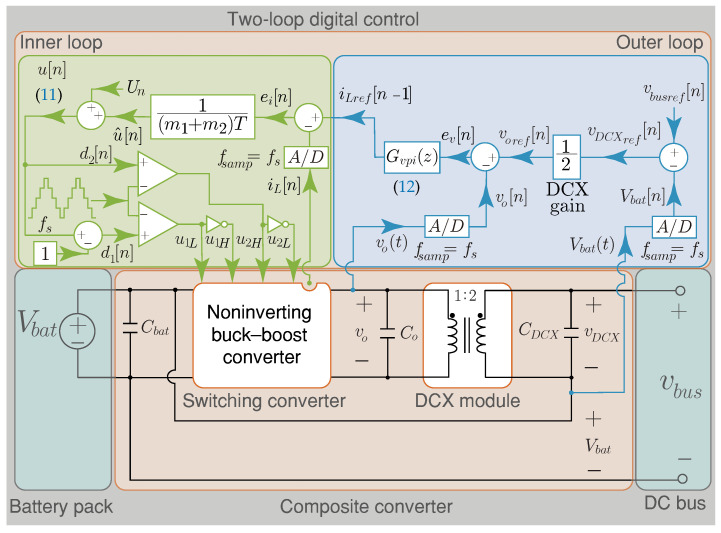
Schematic diagram of the composite converter control method.

This section presents the control design of the composite converter. This strategy consists of two nested control loops with an inductor current (inner loop) controller, based on a discrete-time sliding-mode current control (DSMCC) and a capacitor voltage (outer loop) controller based on a simple PI control as shown in Figure 5. The outer loop regulates the dc bus voltage, considering the DCX module’s gain to give the output voltage reference of the switching converter block $v_{oref}\left[n\right]$. The outer loop provides the output current reference of the converter $i_{Lref}\left[n\right]$ through a Proportional–Integral (PI) control for the inner loop.

### 3.1. Analysis of the Digital Inner Loop

A DSMCC is a robust control with an easy implementation [38]. In this work, this control is extended in a composite structure for the noninverting buck–boost versatile converter. The variable control u[n] is calculated in the *n*-th time sample period that guarantees that the control surface (8) is reached in the next sampling period ($f_{samp}=f_{s}$) [39].

(8) $$s\left[n\right]=i_{Lref}\left[n-1\right]-i_{L}\left[n\right].$$

The current $i_{L}$ has a slope of $m_{1}$ and falls with a slope $-m_{2}$. Table 2 presents the converter current $i_{L}$ waveform slopes based on the equation for $\frac{di_{L}}{dt}$ from (2) for the boost and buck modes.

Assuming the averaged model of the converter’s current output slope $\frac{di_{L}}{dt}\approx \frac{i_{L}\left[n+1\right]-i_{L}\left[n\right]}{T}$, the output current expression in discreet form is:

(9) $$i_{L}\left[n+1\right]=i_{L}\left[n\right]+T\left(m_{1}+m_{2}\right)d_{x}\left[n\right]-m_{2}T.$$

Therefore, the duty cycle expression is

(10) $$d_{x}\left[n\right]=\frac{1}{(m_{1}+m_{2})T}\left(i_{Lref}\left[n\right]-i_{L}\left[n\right]\right)+\frac{m_{2}}{m_{1}+m_{2}}$$

where the mark *x* is different for each operating mode (*x* = 1 for boost mode and *x* = 2 for buck mode), the current slopes $m_{1}$ and $-m_{2}$ correspond with those listed in Table 2, and $i_{Lref}$ is the current reference. For the inner loop, the signal $i_{L}\left(t\right)$ is sampled at the start of each switching period to achieve $i_{L}\left[n\right]=i_{Lref}\left[n-1\right]$ at the end. The steady-state duty cycle is $U=m_{2}/\left(m_{1}+m_{2}\right)$, and the variable control u[n] can be defined as

(11) $$u\left[n\right]=\frac{1}{(m_{1}+m_{2})T}\left(i_{Lref}\left[n\right]-i_{L}\left[n\right]\right)+U_{n}$$

where $U_{n}=U$ for buck mode, and $U_{n}=1+U$ for boost mode. The schematic diagram of the control for the composite converter architectures is shown in Figure 5.

### 3.2. Analysis of the Digital Outer Loop

The control method implemented to regulate the composite converter’s output voltage ($v_{bus}$) is shown in Figure 5. This control consists of a two-loop digital control. The buck–boost converter module allows controlling the DCX output voltage. This strategy enables smooth transitions between buck and boost operations and during the dc bus reference voltage changes. The digital control proposed consists of an inner loop current controller with an outer voltage PI digital control. To guarantee dc bus voltage regulation, it is necessary to add a slower outer voltage control loop. Consequently, the current-controlled buck–boost converter responds quickly to power variations in the load, while the dc voltage control loop compensates voltage variations through constant power by charging or discharging the converter’s output capacitor $C_{o}$. Hence, this PI control is designed to consider the filter output capacitor value $C_{o}$ and the desired loop-gain crossover frequency ($f_{c}$). The voltage reference of the output buck–boost converter is obtained from the bus reference voltage $v_{busref}\left[n\right]$ and the battery voltage $V_{bat}\left[n\right]$, taking into account that the DCX module has a turns ratio ($N_{DCX}$) of two. The converter’s output voltage is measured to calculate the voltage error $e_{v}\left[n\right]$ and provides the current reference $i_{Lref[n-1]}$ as shown in Figure 5. The PI voltage controller in the *z* domain is

(12) $$G_{vpi}\left(z\right)=K_{pv}+\frac{K_{iv}T_{samp}}{z-1}$$

where $K_{pv}=2\pi \;f_{c}C_{o}$, $K_{iv}=K_{pv}/T_{i}$, and $T_{samp}$ is the sample period ($1/f_{samp}$). Therefore, the proportional coefficient ($K_{pv}$) sets the bandwidth of the voltage loop, while the phase margin (PM) is tuned to be higher than $50^{\circ }$, adjusting $K_{pv}$ after setting $T_{i}=10/\left(2\pi \;f_{c}\right)$ for the integral coefficient $K_{iv}$.

## 4. Simulation and Experimental Results

**Figure 6:**
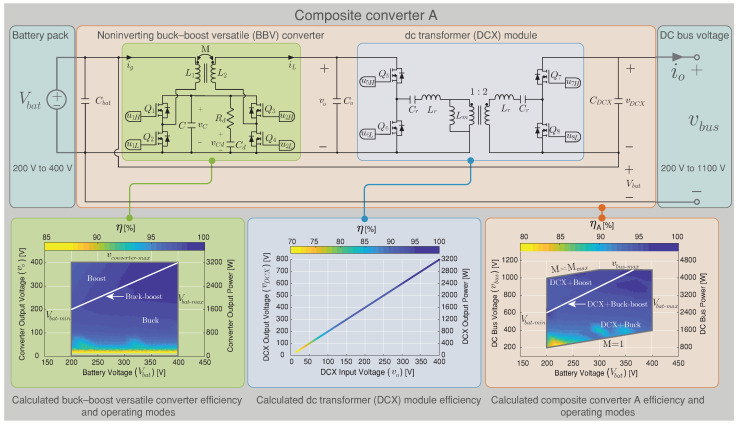
Efficiency results of a composite converter architecture using the BBV converter under different battery and dc bus voltages.

**Figure 7:**
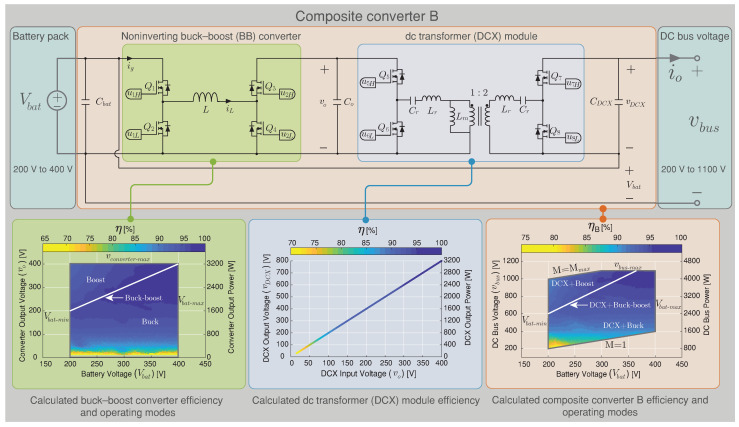
Efficiency results of a composite converter architecture using the BB converter under different battery and dc bus voltages.

In this section, frequency responses are validated with the experimental results of the versatile buck–boost converter prototype. Efficiency power conversion results of the BBV converter as part of a composite architecture (Composite converter A, see Figure 6) and a classical noninverting buck–boost converter (BB) (Composite converter B, see Figure 7) are compared. The experimental and HIL (Hardware in the loop) testing are developed to verify the proposed composite architecture. The HIL test has been split into two subsystems corresponding to the plant and the controller. On the one hand, the plant subsystem corresponds to the composite converter (A based on the BBV converter or B based on the BB converter) and modeled on the PLECS RT Box 1 for HIL evaluating. On the other hand, the controller subsystem is implemented in an inexpensive signal controller (DSC) from Texas Instruments LAUNCHXL-F28069M. The controller consists of a double loop control algorithm to regulate the dc–dc converter’s output voltage (BBV or BB). The BBV converter design was realized following the steps shown in Algorithm 1 for the input parameters: $\Delta i_{L}=1.5$ A, $P_{Rd}=3$ W, for boost mode ($V_{g}=200$ V and $V_{o}=300$ V), T=10 μs, maximum current $I_{L}=8$ A, and $\Delta v_{c}=13$ V. Therefore, the selected components are listed in Table 3.

### 4.1. Validation of Frequency Response

**Figure 8:**
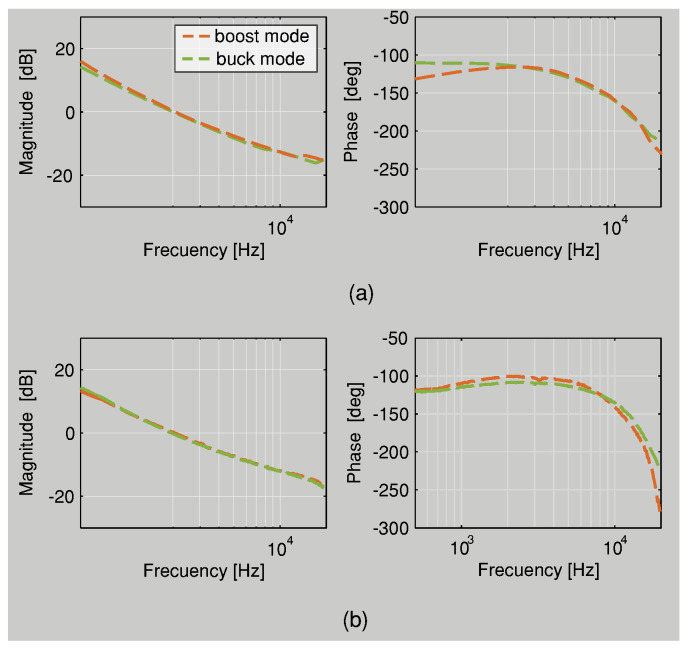
External loop frequency responses of the BBV converter: (**a**) simulated; (**b**) experimental.

The Bode diagram is used to verify the control design of the controllable module. Table 4 lists the experimental and simulated CF and PM for the versatile buck–boost converter, and the Bode plots are shown in Figure 8. The experimental frequency responses are in good agreement with the simulated results, and these conclude that both experimental and simulated values show similar results. The PM values suggest that the system is stable for all operating modes. Therefore, the design control is correct.

### 4.2. Hardware in the Loop (HIL) Validation

**Figure 9:**
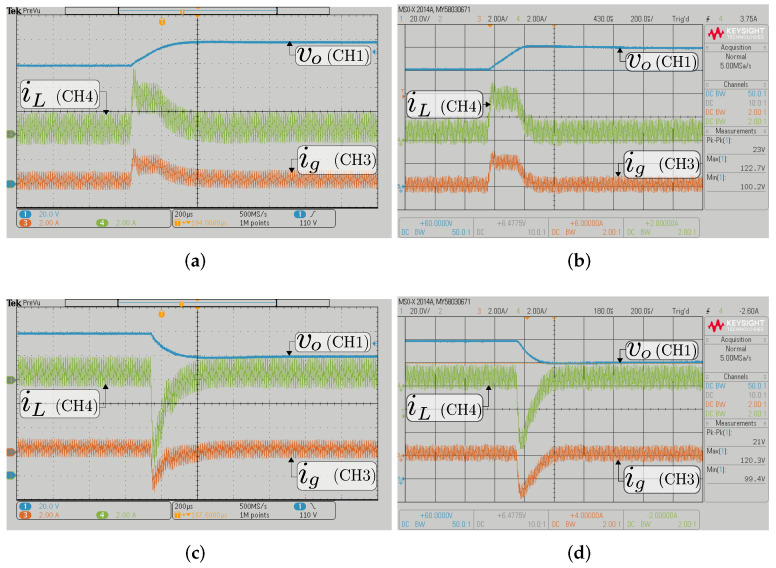
Transient response with a constant resistive load $R_{o}=$ 200 Ω in buck mode ($V_{g}=$ 200 V) for the BBV converter; experimental (**a**,**c**) and HIL (**b**,**d**); transient response when the output voltage reference changes from 100 to 120 V (**a**,**b**) and from 120 to 100 V (**c**,**d**). CH1: $v_{o}$ (20 V/div); CH3: $i_{g}$ (2 A/div); CH4: $i_{L}$ (2 A/div); and time base of 200 μs.

**Figure 10:**
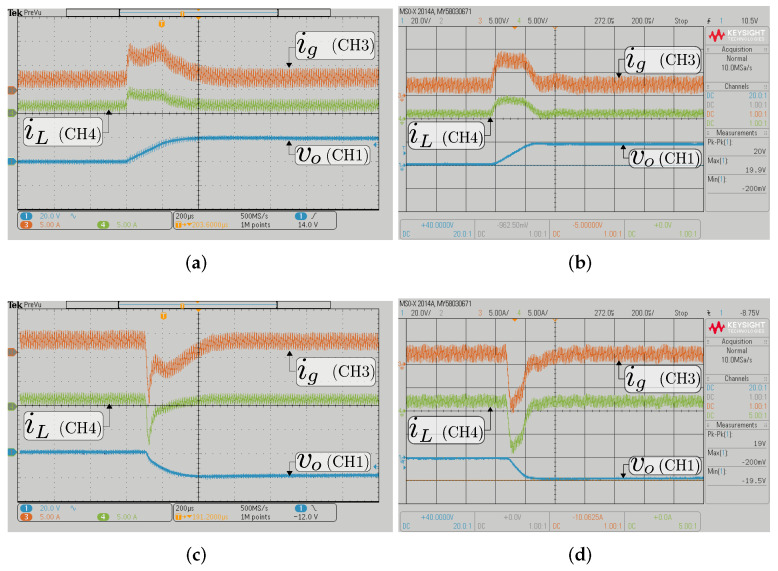
Transient response with a constant resistive load $R_{o}=$ 200 Ω in boost mode ($V_{g}=$ 200 V) for the BBV converter; experimental (**a**,**c**) and HIL (**b**,**d**); transient response when the output voltage reference changes from 294 to 314 V (**a**,**b**) and from 314 to 294 (**c**,**d**). CH1: $v_{o}$ (20 Vac/div); CH3: $i_{g}$ (5 A/div); CH4: $i_{L}$ (5 A/div); and time base of 200 μs.

The double loop control strategy implemented using HIL has been validated with experimental tests. In Figure 9 and Figure 10, the system’s response can be observed when the output voltage reference changes ±20 V in a step form.

The figures show experimental and HIL resulting waveforms of the input, output current, and output voltage when the input voltage $V_{g}=200$ V and a constant resistive load $R_{o}=$ 200 Ω from Figure 4. For buck mode results, Figure 9 illustrates the signal waveforms when the output voltage reference changes between 100 V and 120 V. Note that the output current reference is tracked perfectly. In Figure 10, the results of the converter working in boost mode can be observed, and where the output voltage reference changes between the values of 294 V and 314 V, the results are shown in the ac component to appreciate the output voltage variation in the boost mode. A good agreement between the experimental and HIL results is observed, which validates the correct design of the HIL system.

**Figure 11:**
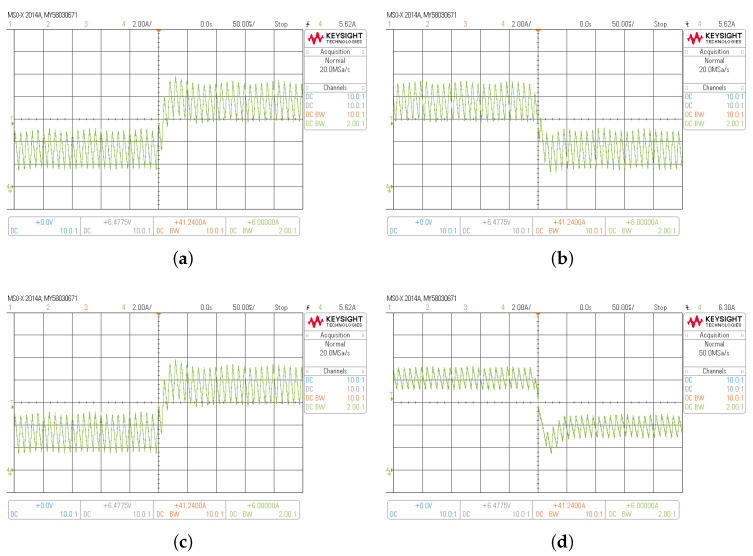
Transient responses of the buck–boost converters current loop DSMCC HIL: (**a**,**b**) in buck mode; (**c**,**d**) in boost mode; from 4 A to 8 A (**a**,**c**); and from 8 A to 4 A (**b**,**d**).

The current loop response for the BBV is shown in Figure 11. This figure shows HIL results with a step change of the current reference of ±4 A for buck and boost mode, respectively. The input voltage is set in 300 V, the output voltage is $V_{o}=400$ V for boost mode and $V_{o}=100$ V for buck mode. As shown, the output current is well regulated. For buck mode, the peak-to-peak output current ripple ($\Delta i_{L}$) is near 3 A, and the output current ripple value is near 1.5 A for boost mode. Thus, the proposed current control strategy performance during current step reference change is also validated.

### 4.3. BBV in Composite Converter

Finally, tests of the BBV converter as part of the composite converter were performed. The rated operating conditions of each module and the composite converter are listed in Table 5.

**Figure 12:**
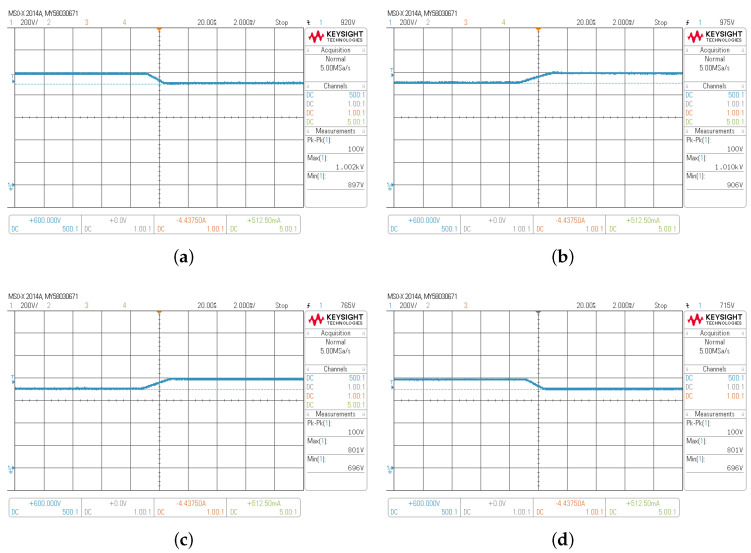
Transient responses of the composite converter output voltage with $V_{bat}=300$ V (**a**,**b**) in boost mode; (**c**,**d**) in buck mode from 1000 V to 900 V; (**a**) from 900 V to 1000 V; (**b**) from 700 V to 800 V; and (**c**) from 800 V to 700 V.

**Figure 13:**
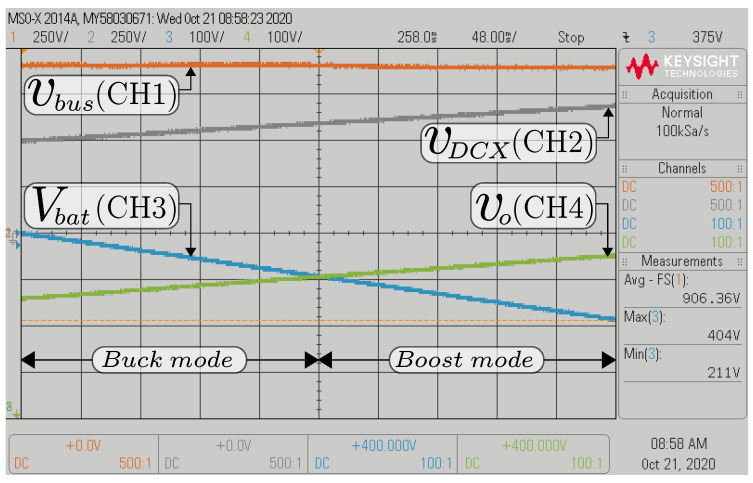
HIL results for the output dc bus voltage regulation of the composite converter A when the battery voltage is discharging.

Transient responses are realized by a step change in dc bus voltage ($v_{bus}$) of the composite converter A (Figure 6) with a $V_{bat}$ of 300 V. The dc bus voltage is changed from 900 V to 1000 V and from 1000 V to 900 V for boost mode in Figure 12a,b, whereas the $v_{bus}$ is changed from 700 V to 800 V and from 800 V to 700 V for buck mode shown in Figure 12c,d. As it is shown, the output voltage followed the reference quickly for both operation modes. Figure 13 shows the dc bus voltage for the composite converter A while the $v_{bus}$ is regulated to 900 V, and the battery voltage ($V_{bat}$) changes from 360 V to 220 V to emulate a discharge. In this case, the buck–boost converter’s output voltage $v_{o}$ and the DCX’s output voltage ($v_{DCX}$) increase when the battery voltage is being discharged to keep the output voltage regulated at 900 V. It can be observed that the dc bus voltage is tightly controlled to its desired value, showing a soft transition from DCX+buck mode to DCX+boost mode.

**Figure 14:**
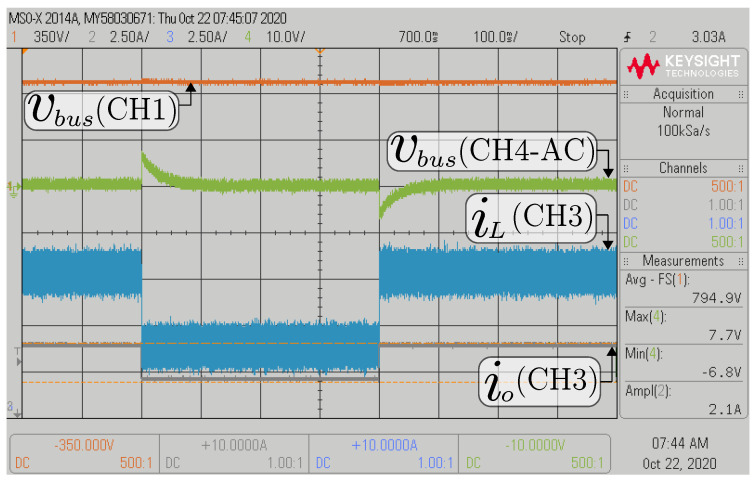
HIL results for the transient response of 50% step change in the current load for buck mode.

**Figure 15:**
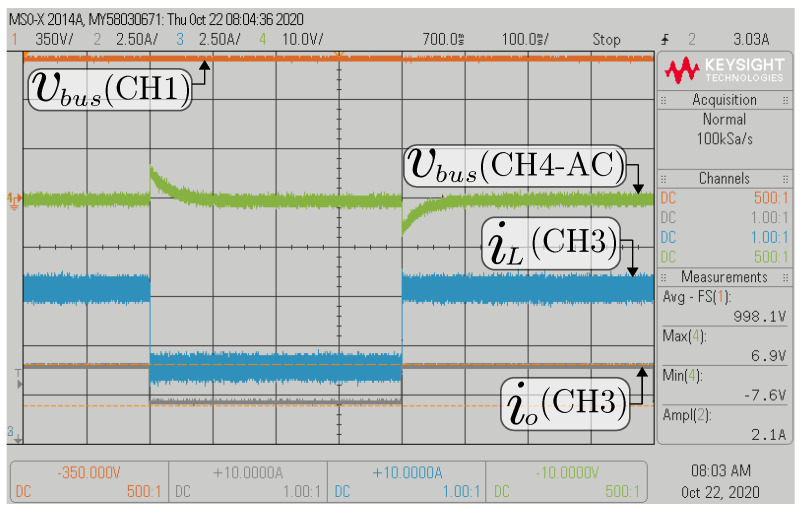
HIL results for the transient response of 50% step change in the current load for boost mode.

Figure 14 and Figure 15 show the transient response front of the 50% step change in the composite converter’s current load. In the case of the buck mode, the dc bus voltage ($v_{bus}$) is regulated to 800 V, and the battery voltage ($V_{bat}$) has a voltage of 300 V while the power load changes between 1600 W and 3200 W (see Figure 14). In the boost mode, the dc bus voltage is regulated to 1000 V, and the battery voltage ($V_{bat}$) has a voltage of 300 V while the power load changes between 2000 W and 4000 W (see Figure 15). In these experiments, an outstanding regulation of the inner DSMCC loop is demonstrated, evidenced by a zero steady-state error in the dc bus voltage.

### 4.4. Efficiency Results Comparison

**Figure 16:**
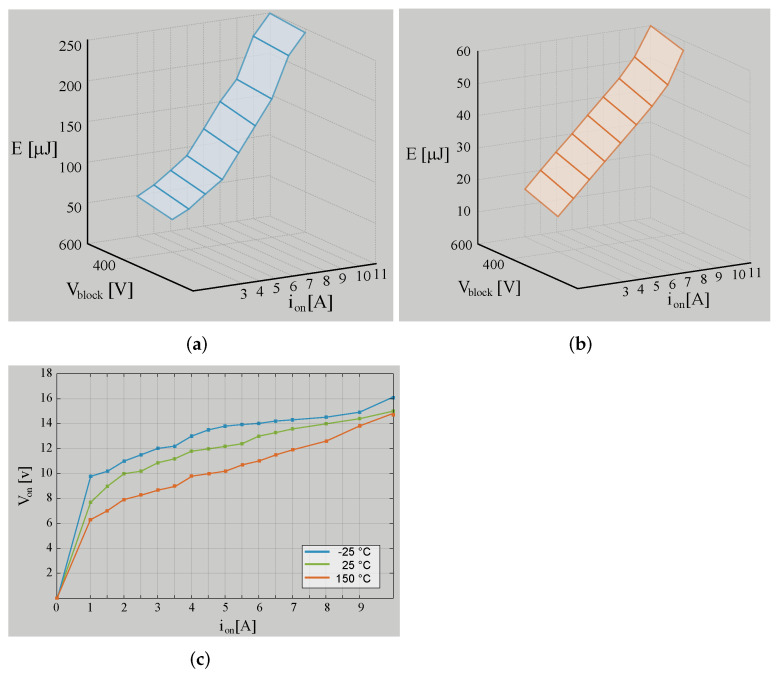
Switching and conduction losses of SCT2450KEC SIC parameters of PLECS: (**a**) turn-on losses; (**b**) turn-off losses; (**c**) conduction losses.

In this section, a comparison has been made regarding the efficiency simulated results between the composite converter using a BBV converter (composite converter A) and the composite converter using a BB converter (composite converter B). The BBV converter for high-voltage application was presented in [28], and its parameters are listed in Table 3. The BB converter was designed under the same characteristic as the BBV converter, and the control strategy presented in Section 3 was implemented for both composite converters. The efficiency results are acquired using the tool PLECS through its thermal model. This simulation is carried out using the heat sink components for the power device SCT2450KEC employed for the dc–dc converters and the DCX module. The conduction, turn-on, and turn-off switching losses are obtained from the datasheet. These were defined as simulation parameters as shown Figure 16.

**Figure 17:**
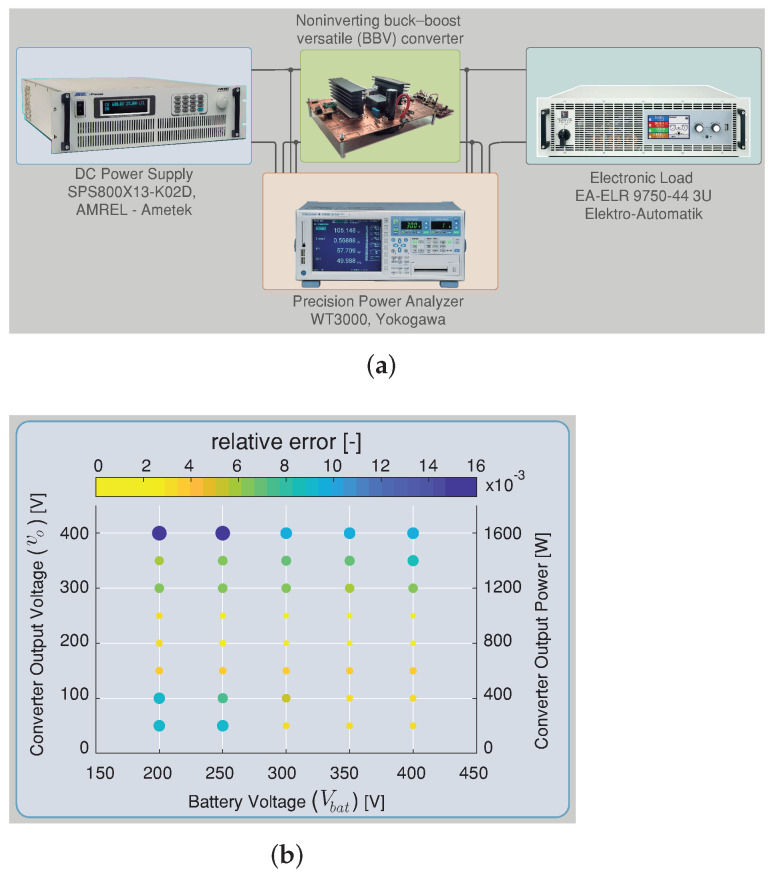
(**a**) Diagram of the experimental setup: dc–dc converter, power analyzer, and electronic load; (**b**) efficiency relative error of the PLECS thermal simulation and experimentally measured efficiency. The circle area is proportional to the relative error.

The thermal simulation for the power conversion efficiency of the BBV converter under different input and output voltages was validated using an experimental measurement with the converter prototype shown in Figure 17a. The thermal model of PLECS was validated with efficiency results presented in [28] for the versatile buck–boost converter. The efficiencies were measured using a Yokogawa WT 3000 precision power analyzer connected at the input and the output of the converter and were taken with the converter working with $I_{L}=4$ A as shown in Figure 17a. Figure 17b calculates different relative error points of simulated and experimental power conversion efficiency results for the versatile buck–boost converter. These results indicate a small relative error between the thermal simulation and the experimental results in all the converter’s operation points. Therefore, the thermal model provided by the PLECS simulation is a correct approximation to compare efficiency between the composite converters A and B. The efficiency results for composite A which is based on the BBV converter are presented in Figure 6, and the efficiency results for composite converter B based on the BB converter are shown in Figure 7. In these figures, the efficiency of the power conversion is included in each module that forms the composite converter (dc–dc converter and the dc transformer) and the overall efficiency for all the possible operation points. As can be seen in Figure 6, the BBV converter has higher efficiency with a conversion ratio close to one $(M_{mbbc}=1$), while for the case of the DCX module, this occurs for an output voltage higher than 200 V. The dc–dc converter was designed for a maximum output current ($i_{L}$) of 8 A and a maximum output voltage ($v_{o}$) of 400 V. The composite converter has a conversion ratio M=1 when the output voltage of the dc–dc converter ($v_{o}$) is 0 V ($v_{bus}=V_{bat}$). The maximum conversion ratio of the composite converter ($M_{max}$) depends on the dc–dc converter’s maximum conversion ratio ($M_{mbbc}=2$) and the DCX turns ratio selected $N_{DCX}=2$. Consequently, the composite converter’s maximum ratio is five ($M_{max}=1+M_{mbbc}N_{DCX}$) as long as it does not exceed the maximum dc voltage $v_{bus}$ corresponding to 1100 V. Figure 6 shows the limitation for all the possible operation points.

**Figure 18:**
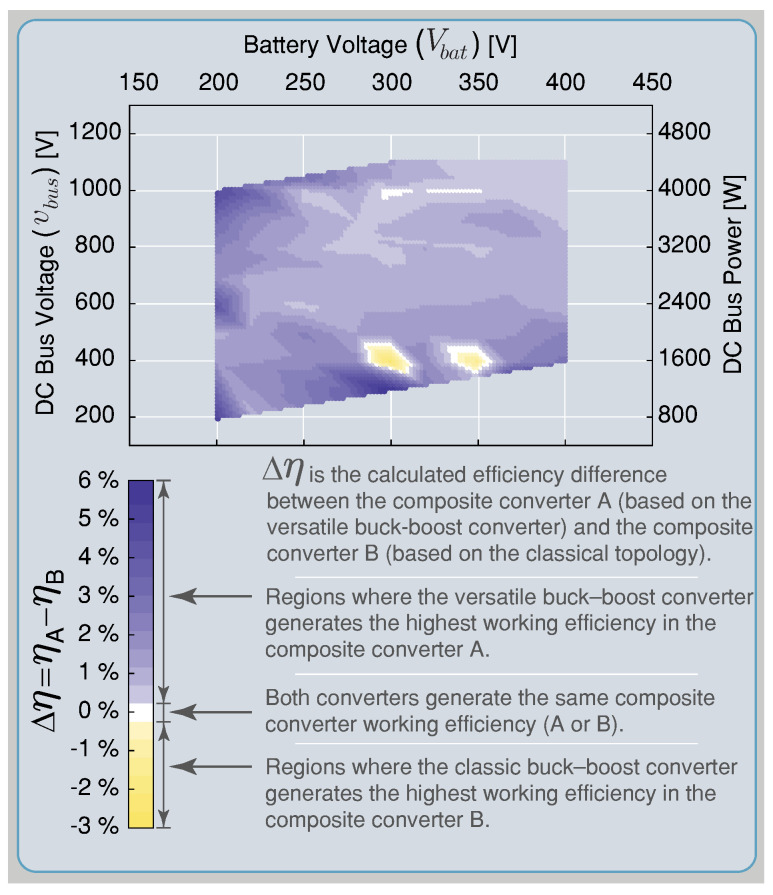
Efficiency difference between the composite converter A and the composite converter B.

**Figure 19:**
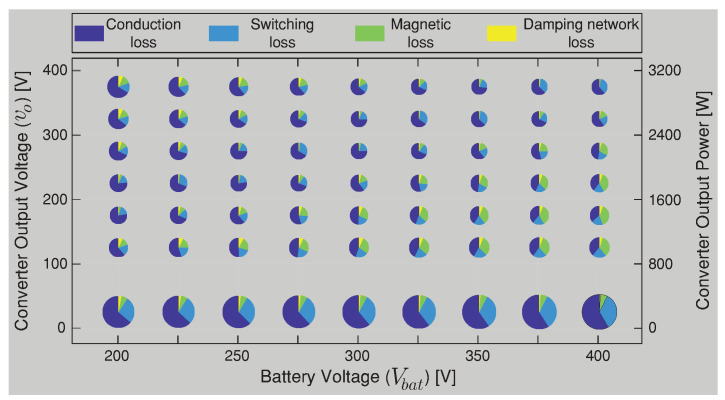
Power loss breakdown in different operating points for the BBV converter.

**Figure 20:**
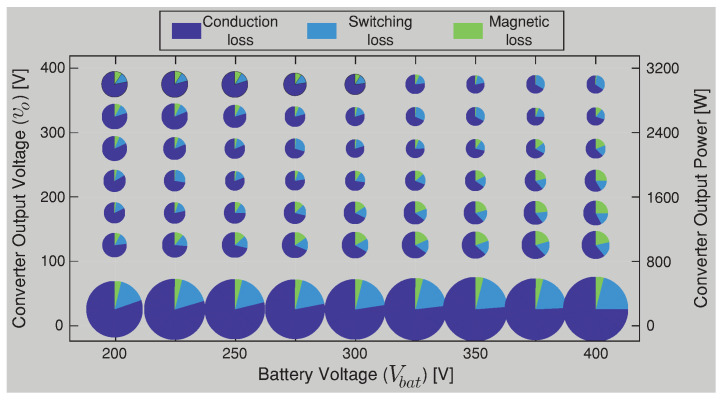
Power loss breakdown in different operating points for the BB converter.

In the composite converter A shown Figure 6, the DCX corresponds to a Half-Bridge LLC resonant converter with a resonant inductor ($L_{r}$) and capacitor ($C_{r}$) in series and with a relatively small magnetizing inductance ($L_{m}$) in the transformer. The DCX module is designed following the step proposed in [40] for an output power of 3.2 kW and a commutation frequency of 100 kHz. The same power conversion efficiency presented for the BBV converter in Figure 6 (composite converter A) was carried out for the composite converter B based on the BB converter shown in Figure 7. The composite converter A achieves the highest power conversion efficiency in the vast majority of operation points as shown in Figure 18, where the efficiency difference between both composite systems ($\Delta \eta =\eta_{A}-\eta_{B}$) is depicted. Outstanding efficiency improvements of up to 6% are achieved if a BBV converter is selected instead of a classical BB one as shown in Figure 18. Power-loss breakdown results for the noninverting buck–boost converters are shown in Figure 19 and Figure 20. These results relate to the overall efficiency results shown in Figure 6 and Figure 7 for the buck–boost converters. In Figure 19 and Figure 20, the size of the pie charts are proportional to the power loss. The power inductor losses are calculated as in [41], and the MOSFET conduction and switching losses are provided by the thermal model of the PLECS simulation. For the BBV converter, the total loss is composed of the MOSFET conduction loss, the MOSFET switching loss, the coupled inductor loss, and the damping network loss. The average value for the results in Figure 19 are 59.6% of power dissipation is associated to conduction loss, 20.6 % to MOSFET switching loss, 15.8% to coupled inductor loss, and 4% to losses in the resistor of the damping network. For the BB converter, the total loss is composed of 73.6% of MOSFET conduction, 9.5% of MOSFET switching, and 16.9% of magnetic loss. From results presented in Figure 19 and Figure 20, it can be observed that the BBV converter has an average percentage of 4.9665% of the total input power in loss for all the operating points compared to 6.4058% of the total input power in loss for the BB converter, demonstrating a higher power conversion for the BBV converter.

## 5. Conclusions

The wide voltage range of the versatile buck–boost converter makes it an appropriate choice in a composite converter architecture for powertrain application. The converter for high-voltage application has been modified by using only one coupled inductor with low parasitic inductance. The output voltage has been regulated to make the dc link voltage variable to improve the system efficiency. The controller implemented for the dc–dc converter is a double loop control, the inner loop has been designed using a discrete-time sliding-mode current control and a PI voltage regulation for the global output voltage. The controller provides a seamless transition between the buck and boost modes. PLECS thermal simulation results confirm that the proposed composite converter improves the efficiency and obtains a superior efficiency result compared to a classical buck–boost composite converter, improving all operating points by up to 6%. HIL results for the buck–boost converter’s dynamic control were validated by experimental results of the versatile buck–boost control. Therefore, HIL results for the composite converter verified the proposed controller’s fast dynamic responses for battery voltage variations and load changes. Thus, the versatile buck–boost converter results are suitable for a composite converter architecture for EV application. Future works will address the use of the versatile buck–boost converter in other known composite architectures.

## Figures and Tables

**Table 1:** Peak-to-peak ripple amplitudes for the BBV converter.

Peak to Peak Ripple	Buck Mode	Boost Mode
$\Delta i_{L}$	$\frac{V_{o}T\left(V_{g}-V_{o}\right)L}{V_{g}\left(L^{2}-M^{2}\right)}$	$\frac{V_{g}T\left(V_{o}-V_{g}\right)M}{V_{o}\left(L^{2}-M^{2}\right)}$
$\Delta i_{g}$	$\frac{V_{o}T\left(V_{g}-V_{o}\right)M}{V_{g}\left(L^{2}-M^{2}\right)}$	$\frac{V_{g}T\left(V_{o}-V_{g}\right)L}{V_{o}\left(L^{2}-M^{2}\right)}$
$\Delta v_{c}$	$\frac{D_{2}I_{L}}{C}T\left(D_{2}-1\right)$	$\frac{D_{1}I_{L}}{C}T$

**Table 2:** Slope of the $i_{L}$ current waveform.

Mode	*m*_1_	−*m*_2_
Buck	$\frac{M\left(V_{g}-v_{c}\right)-L\left(v_{o}-v_{c}\right)}{L^{2}-M^{2}}$	$\frac{M\left(V_{g}-v_{c}\right)-Lv_{o}}{L^{2}-M^{2}}$
Boost	$\frac{MV_{g}-L\left(v_{o}-v_{c}\right)}{L^{2}-M^{2}}$	$\frac{M\left(V_{g}-v_{c}\right)-L\left(v_{o}-v_{c}\right)}{L^{2}-M^{2}}$

**Table 3:** Selected components and parameters for the versatile buck–boost converter.

Parameter	Value or Type
Input voltage $V_{g}$	200–400 V
Output voltage $V_{o}$	100–400 V
Rated Power	3.2 kW
Switching frequency $f_{s}$	100 kHz
Output capacitor $C_{o}$	6x R75PW44704030J, 28 μF, 630 V
Damping capacitor $C_{d}$	MKP1848S62070JP2F, 20 μF, 700 V
Intermediate capacitor *C*	4x R76PN33304030J, 1.32 μF, 630 V
Coupled inductor	*M* = 135 μH and *L* = 270 μH,
	Core: 77,908 Magnetics,
	Number turns: 80,
	Wire size: 18 AWG.
Damping resistance $R_{d}$	2x BPR10100J in parallel, 5 Ω,
	10 W, 500 V
MOSFET Driver	UCC27714D
Power semiconductors $Q_{1}-Q_{4}$	SCT2450KEC

**Table 4:** CF and PM of simulated and experimental voltage loop gain for the BBV.

Operation Mode	Simulated		Experimental	
	CF	PM	CF	PM
	[kHz]	[deg]	[kHz]	[deg]
boost	1.99	63.79	2.03	79.4
buck	1.99	66.52	1.94	71.2

**Table 5:** Composite converter specification.

Module and General Architecture	Parameter	Value
DCX	Max. Input voltage [V]	400
	Max. Output voltage [V]	800
	Max. Output Current [A]	4
	Max. Input Current [A]	8
	Power rating [kW]	3.2
BBV	Max. Input voltage [V]	200
	Max. Output voltage [V]	400
	Max. Output Current [A]	8
	Max. Input Current [A]	16
	Power rating [kW]	3.2
Composite converter	Max. Input voltage [V]	400
	Max. Output voltage [V]	1100
	Max. Output Current [A]	4
	Max. Input Current [A]	20
	Power rating [kW]	4.4

## Data Availability

Not applicable.
